# Sex Differences in Immunity: Implications for the Development of Novel Vaccines Against Emerging Pathogens

**DOI:** 10.3389/fimmu.2020.601170

**Published:** 2021-01-08

**Authors:** Anahita Fathi, Marylyn M. Addo, Christine Dahlke

**Affiliations:** ^1^University Medical Center Hamburg-Eppendorf, 1st Department of Medicine, Division of Infectious Diseases, Hamburg, Germany; ^2^Department for Clinical Immunology of Infectious Diseases, Bernhard Nocht Institute for Tropical Medicine, Hamburg, Germany; ^3^German Center for Infection Research, partner site Hamburg-Lübeck-Borstel-Riems, Hamburg, Germany

**Keywords:** vaccine, hormones, genetic, X-linked gene products, X-chromosome inactivation, miRNAs, emerging infections, sex differences

## Abstract

Vaccines are one of the greatest public health achievements and have saved millions of lives. They represent a key countermeasure to limit epidemics caused by emerging infectious diseases. The Ebola virus disease crisis in West Africa dramatically revealed the need for a rapid and strategic development of vaccines to effectively control outbreaks. Seven years later, in light of the SARS-CoV-2 pandemic, this need has never been as urgent as it is today. Vaccine development and implementation of clinical trials have been greatly accelerated, but still lack strategic design and evaluation. Responses to vaccination can vary widely across individuals based on factors like age, microbiome, co-morbidities and sex. The latter aspect has received more and more attention in recent years and a growing body of data provide evidence that sex-specific effects may lead to different outcomes of vaccine safety and efficacy. As these differences might have a significant impact on the resulting optimal vaccine regimen, sex-based differences should already be considered and investigated in pre-clinical and clinical trials. In this Review, we will highlight the clinical observations of sex-specific differences in response to vaccination, delineate sex differences in immune mechanisms, and will discuss the possible resulting implications for development of vaccine candidates against emerging infections. As multiple vaccine candidates against COVID-19 that target the same antigen are tested, vaccine development may undergo a decisive change, since we now have the opportunity to better understand mechanisms that influence vaccine-induced reactogenicity and effectiveness of different vaccines.

## Introduction

Vaccination has been one of the most successful public health interventions to date. Every year, vaccines prevent millions of deaths worldwide (1). Despite this remarkable achievement, recent outbreaks of emerging viruses such as Ebola, Zika, and the coronaviruses (CoV) SARS-CoV, MERS-CoV, and SARS-CoV-2 underline the importance of a more rapid and systematic vaccine development. In this context, a deeper understanding of host-specific mechanisms that influence vaccine-induced immunity is urgently needed to rapidly develop optimal vaccine strategies for heterogeneous populations.

Classic vaccine development has been rather empirical and employs three vaccine types: i) live attenuated, ii) inactivated, and iii) subunit vaccines. They represent “one bug, one drug” approaches as their respective safety and immunogenicity profiles are pathogen-specific. Their development is both time- and cost-intensive. To accelerate the response to emerging pathogens, so-called “plug-and-play” vaccine platforms have been generated, in which e.g. a carrier system can be adapted to express antigens of interest. These platforms can serve as blueprints to swiftly create vaccine candidates against emerging infectious diseases (EID).

Vaccine development traditionally follows a “one-size-fits-all” approach, although it is well understood that host factors like age, co-morbidities, co-infections, the microbiome and sex influence individual responses to vaccination. With regard to sex, a growing body of evidence indicates its significant role: Women generally develop stronger innate and adaptive immune responses than men (2–4), which can lead to a more rapid clearance and control of infection (5). They often express higher antibody levels and greater T-cell activation, and are thus likely to be more resistant to infections (6). While the trend toward an elevated vaccine-induced humoral and/or cellular immunogenicity in women (7, 8) may be beneficial with regard to efficacy, this may on the other hand lead to increased reactogenicity and negatively impact vaccine safety (6).

Here, we will provide insights gained from clinical trials with regard to sex-specific responses to vaccination and delineate underlying hormonal and genetic mechanisms that are described to affect immunity.

## Influence of Sex on Outcomes of Vaccination

Findings from clinical trials have underlined that sex might have a crucial influence on the vaccine response. A higher magnitude of immune responses in adult women has been observed for a variety of vaccine candidates, including vaccines against influenza, yellow fever, rubella, measles, mumps, hepatitis A and B, herpes simplex 2, rabies, dengue and smallpox (6). Generally, adverse events are more common in adult females than males, which has been shown for influenza, hepatitis B, and yellow fever vaccines (9). The inactivated trivalent influenza vaccine (TIV) has, for example, been described as more reactogenic in women, who reported more adverse events such as injection site pain, myalgias and headaches than men after vaccination (10). Simultaneously, TIV was more immunogenic in women, and half a dose of TIV already induced humoral responses comparable to those in men who received the full dose (10). The importance to take sex into account as a biological variable when assessing vaccine efficacy was further depicted in a phase 3 trial of a glycoprotein D herpes simplex vaccine candidate (8, 11). This vaccine had shown no efficacy in the pre-specified overall analysis of both sexes. However, in a post-hoc sex-stratified analysis, the vaccine was highly protective against genital herpes in females while showing no protective effect in male vaccinees (11). Notably, humoral and cell-mediated immune responses did not differ between sexes, which illustrates the complexity of this issue.

## Non-Specific Effects of Vaccination

Vaccination may also modulate immune responses that are not antigen-specific, and experts have raised awareness for non-specific effects (NSE) of vaccination on immunity. These NSE may impact subsequent morbidity and even mortality from non-vaccine-related infectious diseases. It is assumed that these can be sex-specific (2). In areas where childhood mortality was high, clinical trials of high-titer measles vaccine and subsequent administration of diphteria-tetanus-pertussis or inactivated poliovirus vaccine found that mortality in girls was elevated as compared to boys who received the same vaccination regimen (12). A post-hoc analysis of a phase 3 malaria vaccine trial testing the recombinant protein vaccine RTS,S has also described a sex-differential mortality after receipt of the vaccine (13). Here, an increase in all-cause mortality was observed in girls who had received RTS,S compared to a control group of girls. Those differences were not observed in boys. The results were interpreted with caution and the possibility that they were incidental was raised. The WHO shared this interpretation and recommended the vaccine for use (14), however, others indicate that the findings were highly significant and may be due to NSE and emphasize that the observed safety signals need to be further monitored in phase 4 post-licensure studies, and should also be investigated in preclinical studies (15).

## Viral Vaccines

Findings from clinical trials assessing viral vaccines are of particular interest in the field of EID vaccine research, since an array of recombinant viral vectors are now in development for novel vaccine candidates. These candidates include replication-deficient vectors that deliver the antigenic insert into the cell and thereby induce antigen-specific immune responses. In comparison, replication-competent vectors do replicate and, therefore, may induce a stronger immune response to the vector and tend to be more reactogenic and immunogenic.

Modified Vaccinia virus Ankara (MVA) is a replication-deficient viral vector, has been licensed as a smallpox vaccine (Imvamune^Ⓡ^/Imvanex^Ⓡ^), and has been extensively tested. Males showed on average 27% higher anti-MVA titers (16), and stronger T-cell responses to Dryvax^Ⓡ^, another poxvirus vaccine than females, in addition to statistically significant sex-related differences in interleukin (IL)-2, IL-1β, and IL-10 secretion (17). There was no reported sex difference in adverse events to Dryvax^Ⓡ^ vaccination (18). During the last decade, MVA has been further developed as a promising recombinant viral vector for a multitude of pathogens (19–21). A MVA-based vaccine candidate against MERS-CoV has successfully completed phase 1 testing (22) and the MVA-based filovirus vaccine MVA-BN Filo^Ⓡ^ has been approved in 2020 as part of a heterologous prime-boost regimen against Ebola virus (EBOV) (23). MVA-vectored vaccine candidates are currently also evaluated against Coronavirus Disease 2019 (COVID-19), and the candidate MVA-SARS-2-S has recently entered clinical trials (NCT04569383). So far, these studies have not reported on sex differences.

Adenoviruses (Ad) are widely used as recombinant vaccine vectors and both chimpanzee and human adenovirus vectors, including different serotypes like Ad5 and Ad26, have entered clinical trials (24–27). In a clinical trial of the anti-EBOV vaccine Ad5-EBOV, fever was significantly more prevalent in men than in women (28). For another Ad5-vectored vaccine expressing HIV-antigens, a study that assessed predictors of cellular immune responses against this vaccine revealed that female sex was correlated with a higher number of vaccine responders (29). The most developed viral vector vaccine candidates against COVID-19 are Ad-based (30); these include the chimpanzee adenovirus (ChAd)-vectored vaccine ChAdOx1 nCoV-19, a heterologous Ad26/Ad5-vectored vaccination regimen, as well as an Ad5-vectored candidate; and phase 1/2 (ChadOx1, Ad26/Ad5) and phase 2 results (Ad5) have been published (27, 31, 32). The clinical studies of these candidates all included both sexes and the candidates were found to have an acceptable safety profile and to be immunogenic. Sex-differences could not be inferred from the phase 1/2 trials, which are small by design. However, the phase 2 trial, assessing a single dose of Ad5 vectored vaccine against placebo, included 508 participants with a balanced sex ratio. Humoral and cellular immunogenicity outcomes did not differ between men and women, but fever was more common in women post-vaccination (27).

The yellow fever virus strain 17D (YF-17D) is a live-attenuated virus vaccine that is highly effective against YF, but has a rather reactogenic profile. In rare cases, YF-17D can cause serious adverse events (SAE). Local and early reactions after YF-17D vaccination have been reported more often in women than in men (33). It has been postulated that this observation may be due to an enhanced innate immune response, as a higher number of TLR-associated genes that activate interferon pathways have been found to be upregulated in women post-YF-17D immunization (6). Notably, YF-17D vaccine-associated neurotropic disease (YF-AND) and YF vaccine-associated viscerotropic disease (YF-AVD)—both rare but highly lethal SAE—have occurred more frequently in men than in women (33). The underlying reasons remain to be elucidated. Currently, YF-17D is also under investigation as a carrier for EID vaccines, like the dengue virus vaccine candidate Dengvaxia^®^ (34) and as a COVID-19 vaccine candidate (30).

## Sex-Specific Mechanisms in Immunity

### Hormonal Factors

Clinically observed sex-differential responses to vaccination may partially be explained by hormonal factors. Women exhibit higher levels of estrogen and progesterone, while testosterone is more highly expressed in men, and circulating hormones are assumed to play a relevant role in immunity (35). Sex steroid hormone levels do not only vary greatly between the sexes, but also throughout the life span. The quality and magnitude of immune response may, therefore, vary between men and women, pre- and post-menopausal women, or adults compared to children. Pre-menopausal adult women generally have stronger immune responses than children, men or women during the post-menopause (36). Hormonal levels also fluctuate during the menstrual cycle: estrogen levels increase during the follicular phase, and progesterone remains low, while the luteal phase is characterized by high estrogen 17ß-oestradiol (E2) and progesterone plasma concentrations (37). While data are scarce, there is evidence that the menstrual cycle might affect immune cell numbers and modulate their activity throughout the 4-week period (38). Regulatory T-cells have been observed to be expanded during the follicular phase (38), while B-cell numbers and activity might increase in the periovulatory period. Monocyte numbers and their Tumor Necrosis Factor (TNF)α production increase during the luteal phase, while their IL-1 levels develop in opposing fashion. Data on Natural Killer (NK) cell number and activity have been conflicting, but a study by Souza and colleagues observed no correlation between progesterone and NK cell activity, while cytotoxicity was higher in the follicular than in the luteal phase of the menstrual cycle (38). However, the question whether and to what extent hormonal fluctuations are relevant for the vaccine response has not been comprehensively investigated so far.

The mechanisms of sex steroids that influence immune responses are generally driven by their binding to receptors, which in turn directly influence pro- and anti-inflammatory signaling pathways (39). Numerous immune cells express estrogen (ERs), androgen (ARs) and progesterone receptors (PRs) to varying degrees. The binding of sex steroids can directly affect the immune cell. Estrogens have been implicated in plasmacytoid dendritic cell (pDC) homeostasis, which is a key cell in antiviral immunity. pDCs produce large amounts of interferon (IFN)-α in response to a wide range of viruses, but also other microbial stimuli (40), and probably vaccines (41, 42). The induction of type I IFN drives the activation and anti-viral effector functions of immune cell populations and plays a pivotal role in inducing adaptive immunity. Human female peripheral blood mononuclear cells (PBMCs) and pDCs produce significantly more IFN-α in response to viral nucleic acids or synthetic Toll-like receptor (TLR)7 ligands than PBMCs and pDCs in men (43, 44). The precise functional mechanisms by which sex hormones regulate the IFN-α response of pDCs are unknown, but are thought to involve ERα signaling (45), and an increased level of estrogen may lead to an increased amount of TLR7-mediated IFN-α secretion by pDCs (46).

The E2 has a strong influence on the functional activity of innate immune cells, and it can greatly influence the quality and extent of adaptive immune responses (47). Elevated E2 levels strengthen type 2 helper T-cell (Th2) reactions, augment humoral immunity and regulate pro-inflammatory responses (5, 6, 48). In comparison, low E2 concentrations may result in Th1-type and cell-mediated responses (5, 49). Notably, E2 levels correlated with the number of antibody-secreting cells and antibody levels, as they are highest before ovulation in females (50). An *in vitro* study could further show that estrogen enhanced antibody production and increased the survival rate of B-cells (51).

In comparison to estrogen, several studies reported that testosterone induces rather immunosuppressive effects. The Y-chromosome includes the sex-determining region (SRY), which is responsible for gonad development and the main driver for higher levels of androgens such as testosterone in men. This immunosuppressive effect has been observed in the context of the seasonal TIV. Higher serum testosterone concentrations correlated with reduced neutralizing antibody responses following influenza vaccination (52). In particular, males with elevated levels of serum testosterone and high expression of genes participating in lipid metabolism were significantly less likely to respond to TIV.

### Genetic and Epigenetic Factors

Sex differences in vaccination can already be observed in children, which suggests that not only hormonal but also genetic and epigenetic factors may influence outcomes of vaccination. For example, vaccines against hepatitis B, diphtheria, pertussis, pneumococcus, rabies, malaria and human papillomavirus induced a greater immune response in female than in male children (3).

An important effect is mediated through the gene dosage between male and female cells. Dosage compensation for X-linked gene products may occur via random epigenetic silencing of one of the two X-chromosomes in females. However, 15-23% of X-linked human genes escape X-chromosome inactivation (XCI) resulting in simultaneous expression of both alleles (53). Since many genes encoded on the X-chromosome regulate immune functions, including TLR7, TLR8, IL-2, IL-3, Forkhead-box-P3 (FOXP3), and C-X-C chemokine receptor 3 (CXCR3), distinct gene expression levels may influence immune- and hence vaccine-specific responses (54).

Interestingly, the key immune receptor TLR7 is linked to the pathophysiology of the autoimmune disorder systemic lupus erythematosus (SLE) (54). In addition to pDCs, TLR7 is also present on monocytes/macrophages and B-cells. The gene encoding for TLR7 can escape from XCI and be biallelically expressed on B-cells, which then display higher TLR7-driven functional responses. This may increase susceptibility to TLR7-dependent autoimmune syndromes in females.

In a mouse model for leishmania infection, biallelic expression was likewise observed for CXCR3. XCI escape led to increased levels of CXCR3 in T-cells that consequently produced more IFN-γ, IL-2, and expressed more CD69 compared with T-cells that expressed CXCR3 only from one allele (55). XCI escape by CXCR3 potentially contributes to enhanced Th1 responses in females, which may affect the sex-associated bias observed during leishmania infection (55). Notably, the importance of the CXCR3 ligand CXCL10 (IP10) in response to a vaccine has been shown by our group in a first-in-human trial testing the recombinant viral vector vaccine against EBOV rVSV-ZEBOV (now renamed VSV-EBOV; tradename: Ervebo^®^) (56), as increased CXCL10 levels in the blood correlated with higher antibody titers. Whether a sex-bias toward CXCL10 in VSV-EBOV vaccinees exists is unknown, as studies with large cohorts including a balanced male:female ratio to evaluate innate immune responses and CXCL10 expression have not yet been performed.

Sex-differential expression of microRNAs (miRNAs) represents another gene-related factor that affects immunity. The X-chromosome contains 10% of the 800 miRNAs, whereas the Y chromosome encodes for only two miRNAs (57). MiRNAs are important regulators of messenger RNA (mRNA) stability and translation, and it is assumed that expression of about 60% of protein-coding genes are regulated by miRNAs (58). In the last decade, the role of miRNAs on the innate and adaptive immune response has been discussed extensively [reviewed in (59)]. Due to the high density of miRNAs encoded by the X-chromosome, it can be concluded that females may express more miRNAs due to incomplete XCI. The expression level of miR-223, for example, which is encoded on the X-chromosome, differs between men and women, either due to a skewed inactivation or an escape of gene silencing. High levels of miR-223 can limit recruitment of innate immune cells due to downregulation of CXCL2 and CCL3 and thereby modulate the magnitude of downstream adaptive immune responses (60). Of note, in a study that evaluated measles vaccination, the expression of miR-223 in B-cells was correlated to the induction of neutralizing antibodies, highlighting the potential impact of miRNAs on vaccine efficacy (61).

## Discussion

Hormonal and genetic differences between men and women might be the main drivers of sex-specific responses to vaccines affecting safety, immunogenicity and efficacy. Results from clinical trials, however, generally may not explain the factors that account for observed sex-specific differences due to their non-mechanistic nature and bear significant limitations, as they often originate from post-hoc analyses, and include a heterogeneous group of vaccine products and regimen.

**Figure 1:**
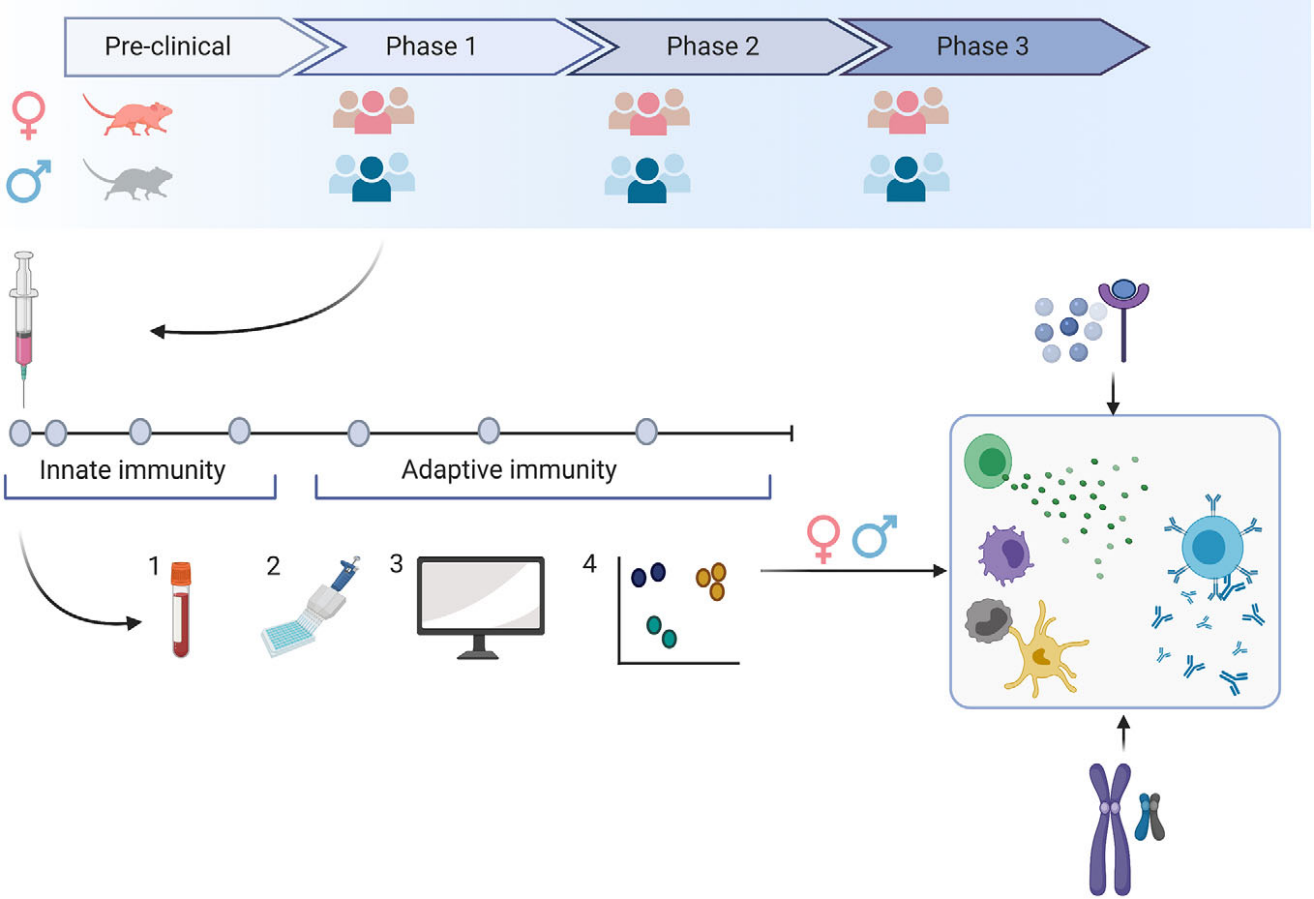
Sex-specific immune responses can lead to vaccine responses that differ in safety, immunogenicity or efficacy. To evaluate the impact of sex on the specific vaccine candidate, we need to include both sexes as early as during the preclinical development stage and aim for a balanced sex ratio in clinical phase 1-3 trials. A detailed snapshot of immune responses to the vaccine can be achieved by frequent blood sampling following vaccination (1) and the application of various technologies (2). Here, we can evaluate responses to the vaccine on the transcriptome, epigenome and proteome level. Using bioinformatic tools (3), we may gain a comprehensive insight into multiple levels of immune responses that may be different in men and women (4). By comprehensively studying innate and adaptive immune responses in men and women, we may better understand how genetic or hormonal differences affect the number and functionality of immune cells.

The importance to gain insight into optimal vaccination strategies for men and women has been increasingly recognized and was recently addressed by the European Union’s Horizon 2020 expert group that emphasized the need to analyze sex-specific side effects in the context of SARS-CoV-2 vaccine research (62). In EID vaccine development, the investigation of sex-differential outcomes of vaccination can be challenging, as early clinical trials evaluate vaccines in small and often homogeneous groups and little data exist on sex-specific immunity induced by the novel platform technologies. However, with more than 200 vaccine candidates against SARS-CoV-2 currently underway, of which over 45 have advanced to clinical trials—up to phase 3 (30) – we now have the unique opportunity to comprehensively investigate sex-specific immune responses induced by various vaccine candidates in pre-clinical and clinical trials (**Figure 1**). In this context, the impact of hormonal cycling on vaccine response should be further investigated.

**Figure 2:**
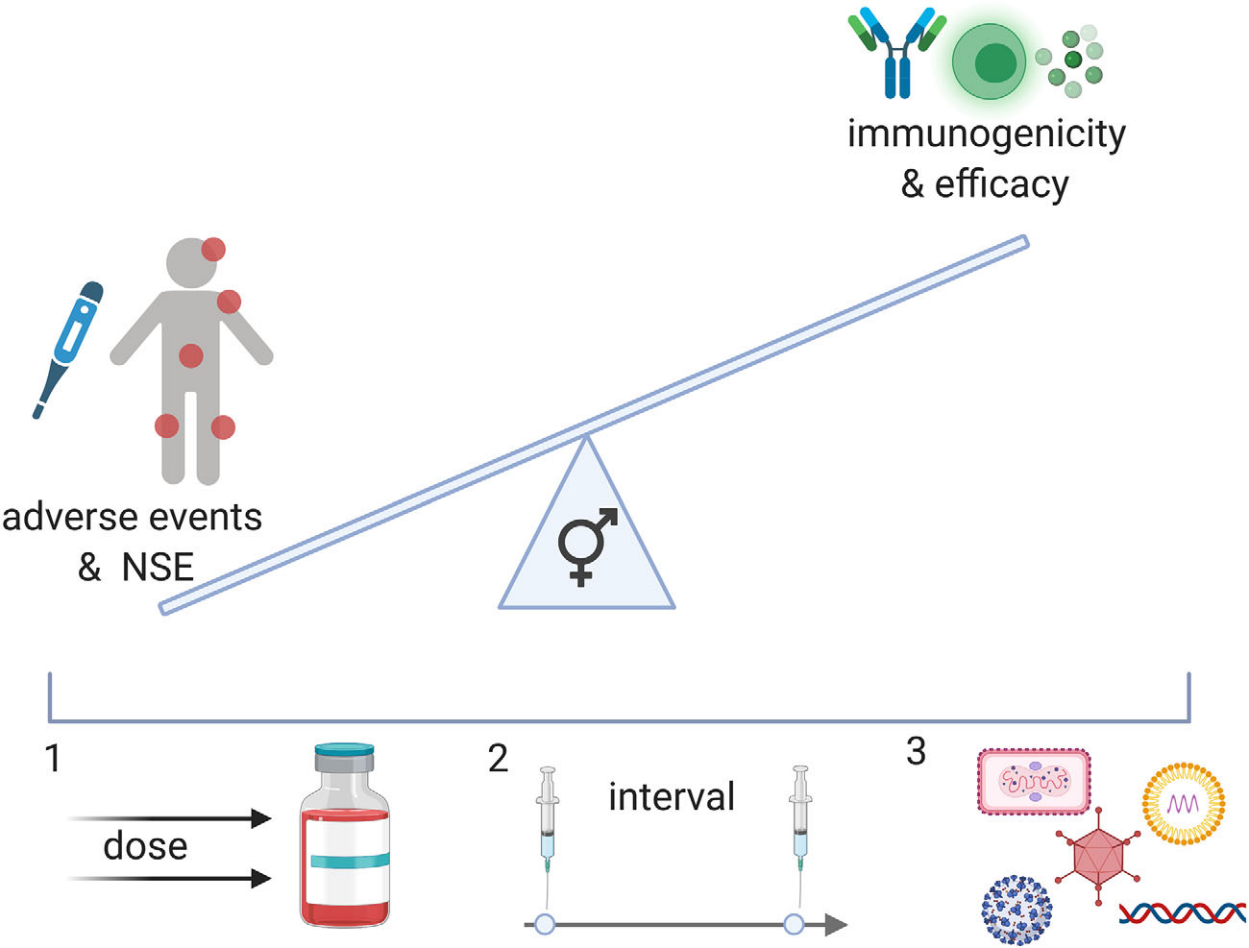
A better understanding of the effect of vaccines in men and women can result in a balanced vaccine response. While NSE of vaccination may improve immune responses to pathogens, negative effects on immunity have also been described in females in specific contexts. In addition, reactogenicity is generally increased in females and may affect the safety profile of vaccines for women and girls. Immunogenicity, however, seems to also generally be increased in females and may therefore affect vaccine efficacy in this population. To achieve an equally beneficial vaccine for men and women, we may administer different doses (1), different intervals (2) or different vaccine candidates (3) (viral vector, nucleosid vaccines, inactivated viruses, proteins).

Frequent blood sampling during clinical trials and the implementation of an array of technologies such as multiple bead assays, flow cytometry, single cell RNA and transposase-accessible chromatin sequencing, multiplex mass, or chip cytometry will allow us to assess sex-differences at multiple levels like the transcriptome, epigenome, and proteome. We can achieve a detailed snapshot of immune responses upon vaccination (**Figure 1**) and correlate these data with measures of outcome. The findings may then guide future vaccine strategies resulting in potentially different dosage levels, different prime-boost intervals or specific vaccine platforms for men and women (**Figure 2**).

We are now at a critical point in time, with an array of new vaccine candidates on the way and state-of-the-art technologies at hand to evaluate host factors influencing vaccine response. If we use this moment right, we may be able to revolutionize vaccine development and strategically design vaccination regimens for distinct populations. Ultimately, this will enable us to optimize vaccination outcomes for the individual.

## Author Contributions

AF and CD conducted a review of articles and wrote the manuscript. MMA reviewed and edited the manuscript. All authors contributed to the article and approved the submitted version.

## Funding

This work was supported by the German Center for Infection Research (DZIF).

## Conflict of Interest

The authors declare that the research was conducted in the absence of any commercial or financial relationships that could be construed as a potential conflict of interest.
